# Experiences Regarding Use and Implementation of Artificial Intelligence–Supported Follow-Up of Atypical Moles at a Dermatological Outpatient Clinic: Qualitative Study

**DOI:** 10.2196/44913

**Published:** 2023-06-23

**Authors:** Elisabeth Rygvold Haugsten, Tine Vestergaard, Bettina Trettin

**Affiliations:** 1 Department of Dermatology and Allergy Centre Odense University Hospital Odense Denmark; 2 Faculty of Health Sciences University of Southern Denmark Odense Denmark

**Keywords:** artificial intelligence, AI, computer-assisted diagnosis, CAD, dermatology, diagnostic tool, FotoFinder, implementation, interview, melanoma, Moleanalyzer Pro, total body dermoscopy, TBD

## Abstract

**Background:**

Artificial intelligence (AI) is increasingly used in numerous medical fields. In dermatology, AI can be used in the form of computer-assisted diagnosis (CAD) systems when assessing and diagnosing skin lesions suspicious of melanoma, a potentially lethal skin cancer with rising incidence all over the world. In particular, CAD may be a valuable tool in the follow-up of patients with high risk of developing melanoma, such as patients with multiple atypical moles. One such CAD system, ATBM Master (FotoFinder), can execute total body dermoscopy (TBD). This process comprises automatically photographing a patient´s entire body and then neatly displaying moles on a computer screen, grouped according to their clinical relevance. Proprietary FotoFinder algorithms underlie this organized presentation of moles. In addition, ATBM Master’s optional convoluted neural network (CNN)-based Moleanalyzer Pro software can be used to further assess moles and estimate their probability of malignancy.

**Objective:**

Few qualitative studies have been conducted on the implementation of AI-supported procedures in dermatology. Therefore, the purpose of this study was to investigate how health care providers experience the use and implementation of a CAD system like ATBM Master, in particular its TBD module. In this way, the study aimed to elucidate potential barriers to the application of such new technology.

**Methods:**

We conducted a thematic analysis based on 2 focus group interviews with 14 doctors and nurses regularly working in an outpatient pigmented lesions clinic.

**Results:**

Surprisingly, the study revealed that only 3 participants had actual experience using the TBD module. Even so, all participants were able to provide many notions and anticipations about its use, resulting in 3 major themes emerging from the interviews. First, several organizational matters were revealed to be a barrier to consistent use of the ATBM Master’s TBD module, namely lack of guidance, time pressure, and insufficient training. Second, the study found that the perceived benefits of TBD were the ability to objectively detect and monitor subtle lesion changes and unbiasedness of the procedure. Imprecise identification of moles, inability to photograph certain areas, and substandard technical aspects were the perceived weaknesses. Lastly, the study found that clinicians were open to use AI-powered technology and that the TBD module was considered a supplementary tool to aid the medical staff, rather than a replacement of the clinician.

**Conclusions:**

Demonstrated by how few of the participants had actual experience with the TBD module, this study showed that implementation of new technology does not occur automatically. It highlights the importance of having a strategy for implementation to ensure the optimized application of CAD tools. The study identified areas that could be improved when implementing AI-powered technology, as well as providing insight on how medical staff anticipated and experienced the use of a CAD device in dermatology.

## Introduction

### Background

**Figure 1:**
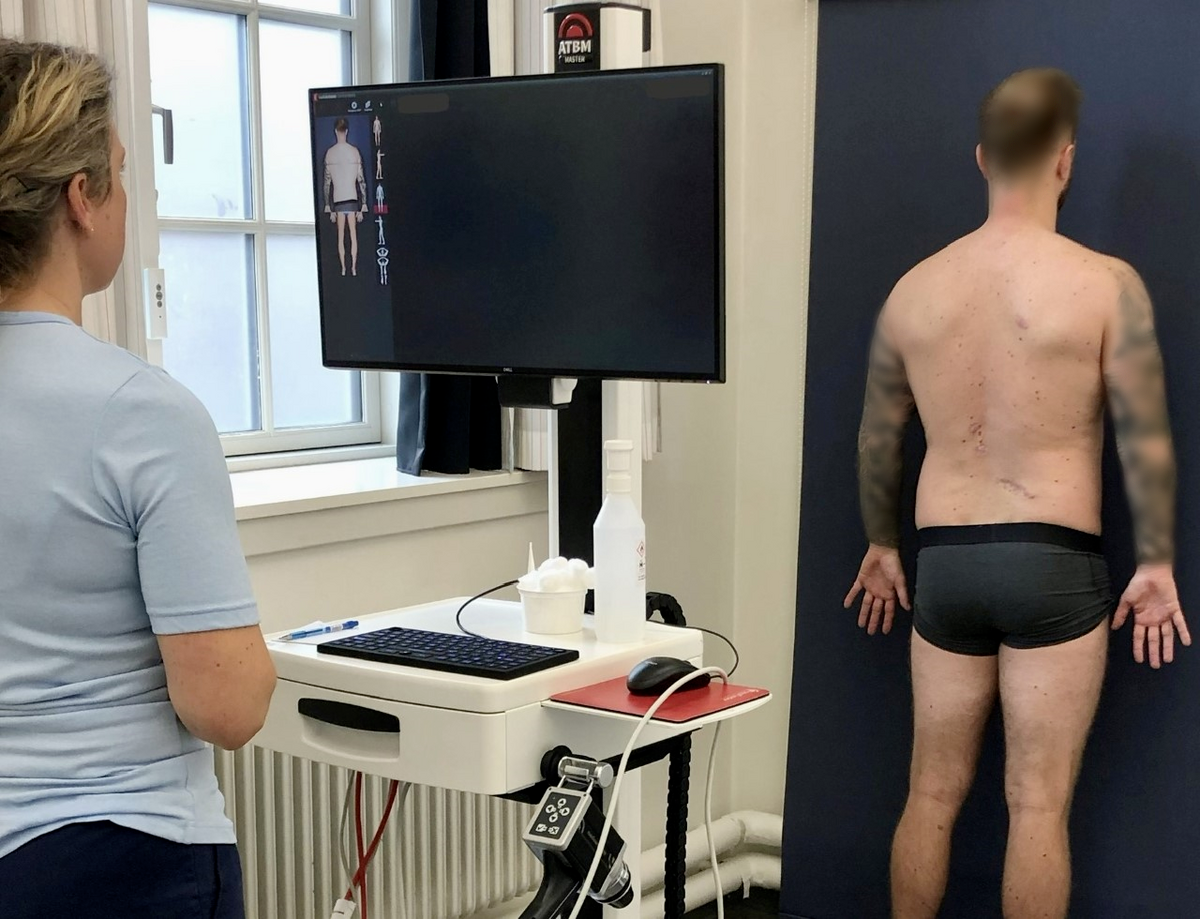
ATBM Master automatically takes photos of a patient’s entire body. The images can be used for future reference, to more easily detect changes in the skin (image shown with patient’s consent).

Artificial intelligence (AI) can be defined as the use of technology and computer algorithms to perform assignments that normally require human intelligence [1,2]. AI is increasingly applied in numerous medical fields [3]. In dermatology, AI can be used when assessing and diagnosing suspicious skin lesions [4]. Such AI-powered methods are known as computer-assisted diagnosis (CAD) systems, and they are capable of analyzing images of the skin and subsequently recognizing malignant lesions [5]. FotoFinder (FF) is a multifunctional imaging platform that implements this CAD technology [6]. FF comprises various units adaptable for disorders related to skin, hair, and nails [7]. One such unit is the ATBM Master (Figure 1), which is optimized to detect and document changes in moles and other skin lesions [8]. This mobile system consists of an upright pole mounted with a screen, a moveable camera, a video dermoscope, and a Q-processor to power it all [9]. Together, these components can execute total body dermoscopy (TBD) [6]. TBD is an automatic way of performing the 2-step method of digital follow-up (DFU) [6]. DFU is a common approach when assessing for skin malignancy and involves taking photos of a patient´s entire body, known as total body photography (TBP), and subsequently digital dermoscopy [10].

**Figure 2:**
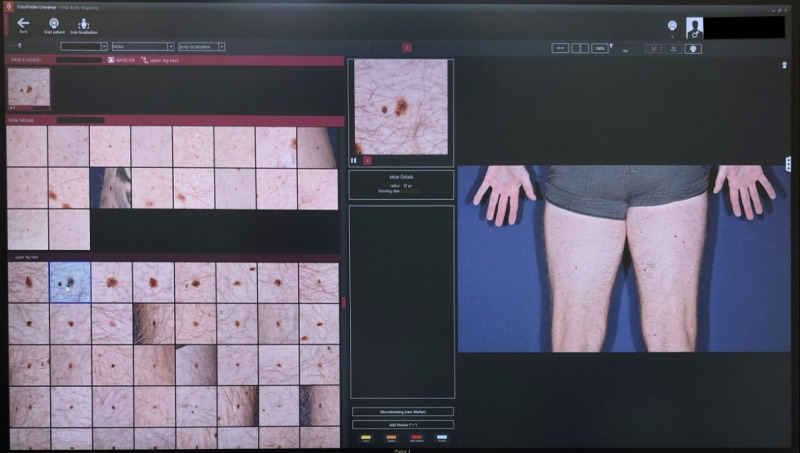
Mosaic View (left), close-up image (center) and overview (right).

Thus, ATBM Master´s TBD module can automatically take photos of a patient from head to toe [9] to obtain a baseline image in which changes in skin lesions can be recognized at successive checkups [10]. Next, after minimum 2 TBP sessions, this CAD system can organize moles according to their clinical relevance and display them on the screen in a so-called mosaic view [9] (Figure 2). In the mosaic view, moles can be neatly arranged into 3 groups, namely new, changed, and unchanged moles [6].

The incidence of skin cancer, both melanoma and other types, is increasing all over the world [11]. Early diagnosis is crucial to improve prognosis [10] and enhance survival of melanoma patients [5]. As over two-thirds of melanomas [12], a potentially lethal skin cancer [5], arise de novo, and about one-third of melanomas develop from preexisting moles [12], TBD can aid in the early identification of harmful changes and malignancy [6]. In particular, TBD may be a valuable tool for the clinician in the follow-up of patients with high risk of developing melanoma, such as patients with multiple atypical moles [13].

The technology behind this includes both the ATBM Master’s individual components and the underlying AI-powered CAD techniques [9]. The camera takes high-resolution, raw-processed polarized photos with a xenon flash [9] and makes use of intricate image modifications [6]. Because of this, the camera system is able to reveal dermoscopic structures when the moles are displayed on the screen [6]. Proprietary FF algorithms are used to identify lesions from the TBP and organize them in the mosaic view [9].

Moreover, ATBM Master’s optional Moleanalyzer Pro software can be used to further assess the moles [9]. This software can give valuable information on lesion parameters such as colors, symmetry, networks, and size [14]. Then, based on these structural attributes, Moleanalyzer Pro can generate a so-called AI score [15] estimating the probability of malignancy of the lesion [16]. Moleanalyzer Pro uses deep learning algorithms [15], more specifically convolutional neural networks (CNN) [17]. This architecture has proven to be suitable for computer-based classification of images, as its multiple neural layers serve as filters detecting the presence or absence of certain features, which in turn determines whether an image should be binary classified into, for instance, benign or malignant [3].

The CNN architecture used in Moleanalyzer Pro is based on a modified version of Google’s Inception v4 [17]. The system comprises 27 layers and was trained and tested through dermoscopic pictures collected from both the International Skin Imaging Collaboration’s (ISIC) dermoscopic archive and several collaborating dermatologists [17]. The broad source of images used for training ensured that the possibility of overfitting was reduced [17]. To validate the CNN’s generalizability, 2 open, external image databases were additionally assessed, namely the Memorial Sloan Kettering data set and ISIC-2018 challenge data set [17]. The performance of this CNN in classifying skin lesions has been compared with that of dermatologists, showing that the two are able to perform on par [17].

Thus, the expanding application of CAD techniques in the medical field [3] could be valuable when responding to medical issues such as skin cancer [18]. However, because the development of many of these systems often focuses on technicalities, rather than practical application in clinical settings, the implementation of such systems may face various obstacles and consequently suboptimal use [19].

### Objectives

To our knowledge, few qualitative studies have been conducted on the topic of implementation of CAD procedures in dermatology. Therefore, the purpose of this study was to investigate how doctors and nurses experience the use of devices having CAD systems, like FF’s ATBM Master unit, in particular the TBD module. In this way, the study seeks to elucidate any barriers to the implementation and application of AI-driven equipment. By discovering these barriers, actions can be taken to ensure a better environment for implementation of additional technologies in the future.

## Methods

### Study Design

The material for the study was obtained through 2 semistructured focus group interviews held at the Department of Dermatology and Allergy Center at Odense University Hospital (OUH). In contrast to traditional interviews, focus group interviews typically consist of 4-8 participants, an interviewer, and an observer [20]. As the goal of this type of interview is to create an interaction and discussion between the participants, in order to explore various perspectives on a certain topic [20], this approach seemed suitable for our purpose.

When preparing the questions for the focus group interviews, the Consolidated Framework for Implementation Research (CFIR) was used. CFIR is a framework designed to orderly assess various aspects of implementation in health care and other disciplines, including any potential barriers [21]. CFIR offers a practical interview guide tool with proposals of relevant questions [22]. Although CFIR encompasses 5 domains [23], this study only focused on three domains: (1) intervention characteristics (FF’s TBD module), (2) inner settings (Department of Dermatology at OUH), and (3) involved individuals (nurses and doctors working with ATBM Master at OUH). Questions about outer settings and processes were left out, mainly due to time constraints for conducting the study. The questions were freely translated from English to Danish.

The device used was the FotoFinder ATBM Master, with Universe software.

The Consolidated Criteria for Reporting Qualitative Research (COREQ) checklist guided the reporting of this study [24].

### Participants

A total of 18 participants, 10 doctors (either dermatologists or dermatology registrars) and 8 nurses, were invited to the focus group interviews by email. Of these, 2 nurses and 2 doctors were unable to attend. Thus, 14 clinicians participated in the interviews. Their working experience in the dermatological field ranged from 6 months to 17 years. The invited participants were chosen because they all regularly worked in the pigmented lesions clinic (PLC), where the ATBM Master unit was commonly used. Thus, all the 14 participants had considerable experience using some functions of the ATBM Master. The TBD module was introduced to the staff at the PLC in June 2021, with subsequent teaching sessions. The participants’ demographics are displayed in Table 1.

**Table 1:** Participants’ demographics.

Category			Participants, n (%)
**Profession**			
	Doctor	8 (57)	
	Nurse	6 (43)	
**Dermatological work experience (years)**			
	0-5	5 (36)	
	5-10	5 (36)	
	10-15	2 (14)	
	>15	2 (14)	
**Gender**			
	Male	3 (21)	
	Female	11 (79)	
**Experience using FotoFinder’s ATBM Master**			
	Yes	14 (100)	
	No	0 (0)	
**Experience using total body dermoscopy**			
	Yes	3 (21)	
	No	11 (79)	

### Data Collection

The focus group interviews were conducted by one of the authors, ERH, and took place in an undisturbed conference room. One more author, BT, was also present and functioned as an external observer taking notes and asking additional questions to clarify issues, if necessary. The 2 interviews took place in October 2021. They lasted approximately 1 hour each, and both were audio recorded and transcribed verbatim.

### Data Analysis

The analysis and interpretation of the interviews were based on the thematic analysis of Braun and Clarke [25]. This framework involves a stepwise process with a thorough acquaintance of the qualitative data, followed by coding, in order to identify patterns within the set of data [25]. Through this approach, vast amounts of data are consolidated into focused topics, available for analysis and interpretation.

Thus, the interviews were transcribed and repeatedly reviewed to increase the accuracy of the transcription. The transcript was read several times in order to further familiarize with the data. Afterward, it was manually coded by one of the authors (ERH). The themes chosen for subsequent analysis were data driven and thus extracted from the data after coding. The findings were discussed among the entire research team. Finally, the particularities of each theme were analyzed when producing the report. The participants were not invited to provide feedback on the findings, mainly because they were all engaged in full-time work in the Department of Dermatology. Any additional involvement in the study would entail them using their spare time, which the authors did not want to advocate.

### Ethics Approval

Before conducting the interviews, the participants were given both written and oral information about the study. All participants signed consent forms. There was no financial compensation for the participation in the interviews.

The study was registered and approved by the Danish Data Protection Agency (21/63319).

## Results

### Overview

The primary aim of this study was to investigate the experiences regarding use and implementation of CAD-based tools such as FF’s ATBM Master unit, in particular the TBD module. However, our research revealed that the participants had less experience with TBD than initially expected. All of them had used parts of the ATBM Master unit, such as its video dermoscope, but during the study it was revealed that only 3 participants had experience using the specific TBD module. In spite of this, all participants were able to provide many reflections and anticipations during the interviews, and 3 major themes and 11 minor themes concerning this topic emerged from the focus group interviews (Textbox 1).

**Overview of the results in this study: major themes and subthemes.**

**1. Organizational matters**

- Lack of guidelines
- Time pressure
- Insufficient training

**2a. Advantages**

- Better monitoring
- Unbiased method
- Faster overview

**2b.** **Disadvantages**

- Low specificity
- Hidden angles
- Poor technicalities

**3. Perspective and expectations**

- Considered a supplement
- Open attitudes

### Organizational Matters

The first major theme that was identified within the data was organizational matters concerning the application of the TBD component.

#### Lack of Guidance

The TBD module had been available in the department since January 2021, but only 3 of the participants had used this function. A lack of guidelines was revealed as the main reason for this. One doctor stated:

I haven’t used it yet because I don’t really know which patients it is intended for.

The participants wanted written instructions with clear definitions and detailed guidance on which patients should be offered TBD, how often these patients should be followed in the clinic, and how to practically perform the TBD function. Most doctors had an idea about which patients they thought were suitable for TBD, namely patients with atypical moles and atypical mole syndrome. However, there were some disagreements about the definitions for these patient groups. As one nurse said:

You have one opinion, but then other doctors have other opinions...

She continued:

The definitions of the various conditions are not clear to all of us, causing us to do things differently.

Due to the variations in definitions and lack of guidance, the participants were seeking somebody to take charge and create universal definitions and guidelines for all of them to follow, to accommodate their uncertainty about TBD’s use. In particular, the “primus motor for such new technologies” was one of the department’s supervising consultants, and the participants awaited this doctor to make the final decision about which patients the TBD module was intended for.

#### Time Pressure

Time was found to be another reason for why the participants were not using the TBD component. A total of 15 minutes were allotted for each consultation. This was perceived as insufficient time to perform TBD, in addition to the standard consultation. One doctor said:

Time is a major limitation to the introduction of new technologies. So, it is necessary to add some time to the consultations, but this has to be organized by the management.

A total of 30 minutes was said to be the minimum time needed to use TBD. However, the participants noted that because they had limited experience using the TBD module fully, this time slot was an estimation.

#### Insufficient Training

Training was yet another organizational matter discovered as being suboptimally addressed. A total of 3 teaching sessions had been arranged in the department since the introduction of TBD. However, due to holidays, meetings, or other reasons, not all participants had taken part in these sessions. When asked about their training preferences, both peer-to-peer training and creating a group of so-called “super users” was requested. Further, the participants wanted to train using the TBD component under small-scale conditions. One nurse explained:

It would be nice to try on some uncomplicated patients, to learn the strengths and weaknesses and get a sense of how the machine works.

A doctor shared this opinion but added:

I need to know which buttons to push and how to save the picture. That is, the technicalities of the machine.

A few of the participants claimed to have enough knowledge to use TBD straight away.

### Advantages and Disadvantages

The advantages and disadvantages experienced and anticipated by the participants when using FF’s ATBM Master and its TBD component were discovered as the second major theme.

#### Advantages

Among the benefits was the ability to compare moles over time. Moles appearing clinically benign upon examination could be revealed as clearly altered when compared with an earlier photo. Changes such as increased size, color deviations, and other irregularities, which otherwise might not have been discovered, could consequently be found. This was especially true in patients with atypical moles, where all moles tend to look irregular. It was perceived as a huge benefit that malignant melanomas could be more easily found in this patient group. One doctor stated:

The fact that the machine can say: this mole used to look like this, but now it looks like that, so it has changed, makes TBD obvious to use.

The unbiasedness of the TBD procedure was another acknowledged benefit. As long as the ATBM Master unit was handled correctly, it should take the same photos regardless of who operated it. Since a variety of doctors worked in the PLC, this feature was anticipated to be helpful in handling the large turnover of doctors.

Lastly, the participants reported that they faster got an overview of the skin, and a pattern among the moles, when the nevi were displayed on a screen. This however, was said to be an advantage of ATBM Master´s screening mode, and not specifically the TBD function.

#### Disadvantages

Among the perceived weaknesses of the TBD component was its low specificity, exemplified by this comment:

It shows an eyebrow or changed lighting as being a new or changed nevus.

This made it challenging to fully trust TBD’s results and made its use very time consuming since the doctors had to sort out structures erroneously marked as new or changed moles. However, the participants claimed that because the TBD module uses AI, it needed to be fed with numerous photos of moles in order to learn to recognize moles and patterns of moles and consequently become better at identifying the malignant nevi.

Other mentioned weaknesses were so-called hidden angles, such as the scalp, behind ears, and genitalia, which the ATBM Master unit was not able to photograph.

The participants also had concerns about the FF device’s user-friendliness and technicalities. When someone used TBD for the first time, one nurse shared the following:

I frequently have to help and assist, until they become familiarized with the machine. Then, the need for help fades out.

Another nurse described how the ATBM Master had missed to photograph part of a patient´s back, causing the nurse not being able to locate a mole of interest. The reason for this was believed to be that wrong height was registered on the patient, causing the TBD to incorrectly adjust the images. This led to frustration as the pictures that had been taken could not be used. A similar example was mentioned about a patient having gained 20-30 kg since the first photographing session, leaving the machine unable to adjust the latter images correctly.

### Perspective and Expectations

The third major theme emerging from the interviews was perspective, referring to the mindset and approach the participants had toward ATBM Master and other new technologies, and expectations, referring to the outlook they had for future use of the TBD module.

#### Supplement

The participants considered the TBD module to be a supplement and broadly agreed that it could not replace the doctor. They believed a manual clinical assessment always should take place, and that the doctors should examine with a dermoscope, even if they used TBD. One doctor said:

If I were to use TBD, I would do the exact same thing as I do today. That is, first thoroughly examine the entire skin with a dermoscope and then compare with an overview photo. Afterwards I would look to see what additional information TBD could possibly give me.

#### Attitudes

The Department of Dermatology’s attitude regarding new technologies was considered open-minded, as it was positive to modern devices and showed a willingness to use resources on purchasing new equipment. For example, the department had 4 ATBM Master machines, compared to other similar hospitals, which only had 2. Some participants, however, described the department as being “too open.” They believed new machines were hastily bought without first ensuring that somebody could operate them, and without first making sure funds were simultaneously put aside for training and implementation. One participant commented:

I feel there is lots of equipment here which is not really used, because time is not set aside for training. When buying a machine, one should also plan for how to implement it.

The individual doctors and nurses also expressed an eagerness toward trying and using the new devices. The COVID-19 pandemic was mentioned as a reason for this openness*,* and it was said that:

After corona, both health care workers and patients have become more open to digitalizations and technologies.

When asked about how the participants experienced the patients’ attitudes toward being photographed and analyzed by an AI-powered device, it was said that patients were pleased with FF’s ATBM Master, and they considered it an additional safety measure.

Regarding the outlook of the TBD module, the clinicians wanted it to sort out irrelevant spots such as shadows and wrinkles. One participant specified what they wanted:

I want it to display, with 100% certainty, lesions with malignant potential and malignant lesions. That would be the ultimate goal when it comes to AI.

## Discussion

### Principal Findings

This study discovered 3 major themes and 11 minor themes regarding the experiences and the implementation of CAD systems such as ATBM Master, with a focus on the TBD module. The first major theme was organizational matters, with lack of guidance, time pressure, and insufficient training being the subthemes. The second major theme was advantages and disadvantages of TBD use, such as improved lesion monitoring and imprecise identification of moles respectively, among others. The third major theme was perspective and expectations, encompassing viewing the TBD module as a supplement and having an open attitude toward implementing new technologies.

### Barriers to Implementation of the TBD Module

Unstructured and inadequate organizational planning has been found to be a hindering factor in the process of AI implementation in health care [26]. For example, the absence of guidelines regarding AI application can cause inconsistent and suboptimal use [26]. This finding aligns well with our study, which discovered that the lack of guidelines concerning use of the TBD module was a major reason for its inconsistent application. This result suggests that the participants were dutiful and wanted to practice their work in a way that was recommended by the department. Moreover, one might speculate that the participants found assurance in knowing they performed their work in the same way as their colleagues.

The absence of designated medical staff in charge of the ATBM Master’s TBD component was identified as another barrier for its implementation. This finding could indicate that the participants felt insecure and sought stability in the fast-paced setting of CAD technology. The presence of a person with executive responsibility for the TBD module could be an assurance for the medical staff, who would have a specific individual to consult on questions about TBD. The term *champion* has been used to describe the existence of such a responsible person taking the lead [26]. For example, in their study about the implementation of AI, Strohm et al [26] described a champion as a person with great interest in the applications of AI and with remarkably good knowledge about its technical aspects. Strohm et al [26] concluded that a champion was a crucial facilitating factor in the initiation and implementation of AI in the organization. A study by Miech et al [27] corroborated this view and identified the existence of champions to be among the elements associated with successful implementation in health care.

This study found that the 15 minutes allotted per consultation were perceived as insufficient time to perform TBD, and that this was an important reason why the clinicians did not use it. To use the TBD module, the participants wanted the consultations to last a minimum of 30 minutes. This finding could imply a worry among the doctors about not being able to perform their work in a satisfactory manner within the given time frame. Time pressure is a common phenomenon among doctors, and executing additional tasks such as TBD, could be perceived as too demanding and subsequently lead to work-related stress [28]. Several studies have found that obtaining full-body photos can be a time-consuming process, as the procedure requires the patient to pose in several positions, and numerous photos are taken [29,30]. A study by Haenssle et al [31] found consultations with patients having over 100 atypical moles, to last up to 60 minutes. This illustrates how some consultations can be very time demanding.

### Experiences and Thoughts Regarding Use of the TBD Module

This study found that the participants considered the TBD module to be a supplementary tool, rather than a replacement for the doctor. In line with this finding, this study also revealed that a major anticipated advantage of TBD was its ability to aid the doctor, especially in the detection and monitoring of subtle changes that could be challenging to find, such as in patients with atypical moles. These results suggest that the doctors trusted their own knowledge, more than they trusted ATBM Master’s TBD module and its technology. At the same time, the participants were open to incorporating this AI-supported CAD device into their clinical work. This could mean they recognized the possibility of the new technology giving them improved diagnostic confidence. A similar result was found by Shen et al [32] in a study concerning dermatologists’ attitude toward AI, which described how AI was considered an assisting tool in the dermatologist’s everyday diagnostic activities. The term augmented intelligence has been used by several to describe this auxiliary role of AI [33,34]. Thus, augmented intelligence illustrates the potent relationship between doctors and technology [34], and some claim this collaboration has a synergic effect [2] resulting in higher diagnostic accuracy [35].

Despite the promising potential of AI-powered CAD systems, this study found that some aspects of the TBD module were currently substandard. A perceived weakness was revealed to be its inability to photograph areas such as the scalp and behind the ears. A similar finding was found in a study by Mar and Soyer [36] about AI for melanoma diagnosis. Their study pointed out that a thorough examination of the skin, including the aforementioned areas, is an important part of a skin cancer consultation [36]. The same study also mentioned how dermatology is a visual, but also a tactile speciality, as some melanomas are best detected upon palpation [36].

Another major weakness was that TBD identified items such as an eyebrow to be a mole. This made it hard for the participants to fully trust ATBM Master’s results and could possibly make them hesitant to use the TBD module. However, AI technology and CAD systems are constantly advancing [18]. In their study, Esteva et al [37] found similar performance levels between dermatologists and certain types of AI when comparing their ability to classify skin lesions, and in this way illustrating how AI has exciting potential for the future.

### Limitations, Strengths, and Future Research

This study has limitations. First, because it was set in Denmark, the results might not be applicable to settings elsewhere. Second, only 3 of the participants had actual experience with the inquired TBD module. Many of the statements were therefore based on the participant’s anticipations. Third, questions about outer settings and processes were left out, leaving these aspects of implementation unexplored. The strength of this study is that it reflected an authentic setting. Just as the focus group interviews that served as the foundation for this study, the dynamic situations encountered in everyday clinical practice are influenced by the various health professions and individuals that take part.

Future research should continue to explore how to implement and make use of CAD systems in ways which could improve patient care. It is important that these new devices are perceived as helpful by clinicians, not a time-consuming burden.

### Conclusions

The use of AI is rapidly expanding in numerous medical fields, including dermatology [3]. However, this study has shown that implementation of new technology does not occur automatically, demonstrated by how only 3 of the participants had used the inquired TBD module.We conclude, that to ensure optimized application of methods using AI, such as CAD systems, a strategy for their implementation should exist. Qualitative studies like ours can provide valuable insight on implementation of AI-supported devices. In this way, better implementation strategies can be created, to make the most of the flourishing potential of AI.

## Abbreviations

AI: artificial intelligence
CAD: computer-assisted diagnosis
CFIR: Consolidated Framework for Implementation Research
CNN: convolutional neural network
DFU: digital follow-up
FF: FotoFinder
ISIC: International Skin Imaging Collaboration
OUH: Odense University Hospital
PLC: pigmented lesion clinic
TBD: total body dermoscopy
TBP: total body photography
